# Parent-Implemented Bedtime Fading and Positive Routines for Children with Autism Spectrum Disorders

**DOI:** 10.1007/s10803-017-3398-4

**Published:** 2017-11-24

**Authors:** Emma Delemere, Katerina Dounavi

**Affiliations:** School of Social Sciences, Education & Social Work, Queen’s University Belfast, 69-71 University Street, Belfast, BT7 1HL Northern Ireland, UK

**Keywords:** Bedtime fading, Positive routines, Sleep, Autism, Parent training

## Abstract

Sleep disorders affect a large portion of those with autism spectrum disorder. Behavioural interventions have been found to increase appropriate sleep behaviours. This study sought to examine the efficacy of two stimulus control interventions (bedtime fading and positive routines) on total sleep duration, sleep onset latency and frequency and duration of night wakings for children with autism using two multiple baseline designs. Secondary dependent variables, namely, educational opportunities, challenging behaviours, parent acceptance and social validity were also analysed. Results suggest some efficacy for both interventions. Increased total sleep duration and decreased sleep onset latency were achieved with bedtime fading. Positive routines showed mixed results with decreased sleep onset latency and increased total sleep duration for two of three participants.

## Introduction

Good quality sleep is vital for cognition, affect, behaviour and life satisfaction (National Sleep Foundation, NSF 2004). A survey conducted in America by the NSF (2004) found that 40 million people suffer some form of sleep disorder. Additionally 60% of adults experience sleep disturbance three or more nights per week. Sleep disorders affect 25–40% of children (Owens 2007), 84% of whom will continue to exhibit them at a 3 year follow-up (Kataria et al. 1987). A longitudinal study conducted by Zuckerman et al. (1987) noted sleep disorders persist throughout lifetime if untreated. Though prevalent, sleep disorder rates increase for those with developmental disorders such as autism spectrum disorder (ASD). Increased sleep onset latency and night wakings, decreased sleep efficiency and variable sleep patterns have been found in comparison to typically developing peers (Vriend et al. 2011). While 20–40% of typically developing peers exhibit sleep problems, this increases to 40–80% for children with ASD (Souders et al. 2009). The National Sleep Foundation identified children with ASD as a high priority population for sleep research (Mindell et al. 2006).

Sleep disturbances negatively impact a variety of daytime behaviours with a reduction of only 30 min hampering functioning (Sadeh et al. 2003). Poor sleep affects cognitive functions such as memory, learning, attention and mood regulation (Gozal 1998), as well as health, behaviour and overall wellbeing (Mindell et al. 2006). Children with ASD appear to suffer additional side-effects such as increased stereotypy, decreased social skills and increased aggressive behaviours (Schreck et al. 2004). Increased stereotypy has been found to be related to anxiety for individuals with ASD (Schlaggar and Mink 2003) suggesting that anxiety may be connected with sleep disorders.

In order to understand why sleep difficulties occur, one must consider biology. All individuals adhere to the circadian cycle of sleep and wake (Finger 1982). Infant sleep differs greatly from adult sleep in that an infant experiences quicker transitions between rapid eye movement (REM) and non-REM phases than an adult (Jin et al. 2013). Electroencephalogram studies show REM sleep precedes arousal and cause wakings (Anders 1979). As a result of increased REM frequency infants are more likely to wake. These cyclic-REM arousals represent a universal vulnerability in all infants which if combined with a maladaptive parenting style (i.e., a style that aids the child in accessing contingencies of reinforcement for sleep incompatible behaviours) make infants prone to sleep difficulties (Anders et al. 1992).

When seeking help for sleep disturbances, often, the first port of call is the paediatrician (Jin et al. 2013). Of paediatricians, 96% feel sleep management falls within their role (Faruqui et al. 2011) while only 18% have received formal training. Medication results from 80% of doctor visits for sleep (Stojanovski et al. 2007). Medication presents a number of limitations, such as poor parental acceptance, long term inefficacy (France et al. 1991), day time hangover effects (Wiggs and France 2000) and poor empirical support (Rosen et al. 2002). Additionally, medication per se does not facilitate access to the environmental contingencies surrounding sleep and as such does not lead to learning of appropriate sleep onset behaviours. One form of medication which has demonstrated some success for children with ASD is melatonin (Cortesi et al. 2010). Melatonin has been found effective at increasing sleep duration and reducing sleep onset latency (Kuhn and Weidinger 2000). However melatonin lacks controlled research findings (Rossignol and Frye 2011). Benefits of medication include its ability to establish sleep as a potent reinforcer (Mindell 1999) and to reduce crying and conditioned vomiting (France et al. 2003). Overall, although there seems to be some evidence to support drug use, this is not sufficient and makes pharmaceuticals unnecessary to obtain desired results.

An alternative to medications are behavioural interventions. A systematic review of behavioural sleep interventions found that 94% of all studies resulted in clinically significant improvements (Mindell et al. 2006). One of the most common interventions is stimulus control, which posits that falling asleep is an operant behaviour reinforced by sleep itself (Bootzin 1977). Falling asleep can be thought of as the final step in an operant behaviour chain (Ferster et al. 1975). This chain commences with pre-bed behaviours and finishes with behavioural quietude prior to sleep onset (Blampied and France 1993). Behavioural quietude allows for internal cues such as sleepiness or tiredness to be discriminated (Blampied and France 1993). Activity acts as a competing response and may interfere with the discrimination of natural cues for sleep onset thus delaying sleep. Halting activities allows exposure to sleep-preparatory discriminative stimuli (SDs) increasing sleep onset likelihood. This perspective is supported by sleep latency data (Carskadon and Dement 1987).

A central assumption of this theory is that if consistent sleep is to occur, steps in the behavioural chain must come under stimulus control of suitable SDs (Bootzin 1977). As is the case with any behaviour, discriminative properties are obtained by stimuli in the environment which reliably signal the availability of reinforcement, in this case sleep. Typical SDs for behavioural quietude are dark lighting, cool temperatures and bedding, the absence of which can lead to individuals experiencing difficulties with sleep onset.

Motivating operations (MOs) are also implicated in sleep onset. MOs such as quality of previous sleep, previous sleep duration and time since previous sleep all alter the reinforcing value of sleep and the likelihood of behaviours which previously gained access to sleep (Michael 1982). As such the value altering and behaviour altering aspects of MOs are important to sleep onset. A number of interventions have been established from this theoretical perspective, two of which hold numerous empirical analyses, positive routines and bedtime fading.

Positive routines consist of a series of pleasurable calming activities undertaken during wakefulness to facilitate sleep onset (Gruber et al. 2011). A routine is defined as a repetitive and observable behaviour pattern (Koulougliti et al. 2008), which contains five to seven activities and takes between 30 and 40 min to complete (Durand 1998). Completion of each step of a routine is praised informing the child of the transition to the next step (Morgenthaler et al. 2006). Activities move from rich to lean reinforcement and from active (e.g. running) to passive (e.g., reading,). Positive routines attempt to establish appropriate sleep onset SDs by establishing a behavioural chain terminating in behavioural quietude. The terminal reinforcement for completing this chain is sleep onset. Studies have posited routine occurrence rather than content is important (Kodak and Piazza 2008). Sleep resumption requires the presence of these sleep associated SDs (Ferber 1990). Thus if these SDs are encountered during wakings, independent sleep resumption is likely. Parents often implement positive routine interventions (Owens 2006). While positive routines are often combined with other intervention components, minimal research to date has evaluated its individual efficacy (Mindell et al. 2006).

Positive routines were first utilised by Milan et al. (1981) as a means to combat negative side effects experienced with extinction. This was found to be effective in reducing night-time tantrums for three children with severe intellectual disabilities within the home setting. However the inclusion of a fading aspect prevents conclusions to be drawn on the efficacy of the positive routines in isolation. A number of follow up studies utilised positive routines within multicomponent interventions (e.g., Adams and Rickert 1989; Knight and Johnson 2014; Christodulu and Durand 2004). Only two studies have evaluated positive routines in isolation. Mindell et al. (2009) evaluated the efficacy of positive routines for 405 typically developing toddlers aged between 7 and 36 months and found them to be effective at reducing sleep onset latency and night wakings. A more recent evaluation by Mindell et al. (2015) examined the impact of routine ‘dose’ on efficacy. This study included over 10,000 typically developing participants and found positive routines to be effective with routine exposure negatively correlating with bedtime difficulties. However both of the above studies involved typically developing participants therefore conclusions cannot be drawn for individuals with ASD.

A second stimulus control intervention is bedtime fading, the central aim of which is to manipulate the sleep wake cycle to increase sleep likelihood. Bedtime fading involves temporarily moving bedtime to more closely coincide with the child’s natural sleep onset, so as to ensure rapid sleep initiation, and then fading this earlier if sleep onset latency remains short according to developmental norms and parental wishes (Morgenthaler et al. 2006). A scheduled wake time is also established and sleep is not permitted outside of these times. This acts to establish sleep as a potent reinforcer. Bedtime fading relies on internal cues of sleepiness caused by sleep deprivation. These serve as establishing operations which increase the reinforcing value of sleep for the child. This facilitates a smooth transition and quick sleep onset, preventing inappropriate behaviours. A central assumption is that sleep resumption following arousal will be facilitated as the SDs associated with sleep onset are readily available (Ferber 1990).

The use of bedtime fading was first shown effective with a 6 year old girl with attention deficit hyperactivity disorder experiencing multiple sleep difficulties (Piazza and Fisher 1989). Piazza and Fisher (1991) also used bedtime fading to increase total sleep duration for two typically developing children within an inpatient facility. The present study was based on Adams and Rickert (1989) which combined positive routines with a simplistic version of bedtime fading. Substantial research into bedtime fading within multi-componential packages has been conducted (e.g., Piazza and Fisher 1991; Ashbaugh and Peck 1998; Christodulu and Durand 2004), while one study has evaluated bedtime fading in isolation (DeLeon et al. 2004) noting an 81% reduction in night wakings and an 82% reduction in self-injurious behaviour for a 4 year old boy with ASD.

While positive routines and bedtime fading have demonstrated efficacy with typically developing children, individual efficacy and efficacy with ASD cannot be established (Mindell et al. 2006). The present study aims to answer three questions. First, it seeks to evaluate if bedtime fading and positive routines are effective in decreasing sleep onset latency and night wakings in young children with ASD. Second, it aims to respond to the question of whether bedtime fading and positive routines are effective in increasing the total sleep duration for young children with ASD. The third question is whether primary caregivers can successfully implement positive routine and bedtime fading interventions. In the same line, the study aims to examine whether these interventions are acceptable to parents.

## Method

### Participants

Six participants were recruited. Criteria for participation were an ASD diagnosis, age between 2 and 7 years, average nightly sleep duration of less than 7 h and an indicated sleep problem on the Sleep Assessment and Treatment Tool. Participants were recruited from an early intervention setting within which the researcher was employed. Within this setting, all participants availed of 20 h per week of one-to-one tutoring based on applied behaviour analysis (ABA). Parent participants noted sleep as an issue for their child and resulted in stress for both child and family. All families who participated were middle class with all parents having completed at least secondary level education. All participants were only children except Mary and Niamh who were twins. All participants obtained their diagnosis from an independent psychologist. Participant details are provided in Table 1. Pseudonyms were used for all participants within the study. The developmental level has been provided based on the most recent evaluation conducted using the Verbal Behavior Milestones Assessment and Placement Program (VB-MAPP; Sundberg 2008). No participants were actively taking medication for sleep difficulties. Participants were randomly allocated to one of the two interventions (positive routines or bedtime fading) with three participants in each.

**Table 1:** Participant demographic data

Participant	Age	Developmental age (VB-MAPP) (months)	Reported sleep issue	Intervention
Mary	6	0–18	Sleep onset Early wakings	Bedtime fading
Thomas	2.5	30–48	Sleep onset Early wakings	
Niamh	6.2	0–18	Sleep onset Early wakings Night waking	
Martin	6.5	0–18	Early wakings Night wakings Sleep onset	Positive routines
John	2.5	0–18	Night wakings	
Alan	4	30–48	Sleep duration	

### Setting

Two settings were used within this study. Consent provision, parent training and interviews were all conducted in an office in the researcher’s place of employment. The second setting was participant homes. All aspects of both interventions were parent implemented within the home setting. All children slept in their own bedrooms as they had prior to the study. Parents reported all participants went to bed in dark or dimly lit rooms (night lights).

### Dependent Variables

Three primary dependent variables were employed. These were (a) sleep onset latency, (b) frequency and duration of night wakings and (c) total sleep duration. Prior to discussing the operational definitions of each, the operational definition of sleep used within this study must be provided. The child is considered asleep when they are lying in bed with eyes closed accompanied by less than one gross motor movement per 30 s and deep breathing (defined as a breathing rate lower than the average breathing rate while awake). Vocalisations may or may not accompany sleep (e.g. snoring, sleep talk, etc.). Sleep offset (waking) is defined as the absence of the above for two consecutive minutes. This definition is broadly consistent with that of Jin et al. (2013).

#### Sleep Onset Latency

Sleep onset latency is defined as the total duration between placing the child in bed and bidding them goodnight, and sleep onset. Sleep onset itself is defined as 1 min of meeting the definition of sleep following 15 consecutive minutes of wake.

#### Night Wakings

Night wakings are operationally defined as the duration between sleep offset (wake) and sleep onset following a 15 min period of continuous sleep. Both frequency and duration were recorded.

#### Total Sleep Duration

Total sleep duration is defined as the total duration between sleep onset and final sleep offset minus total duration of night wakings.

#### Secondary Dependent Variables

A number of secondary dependent variables were also analysed. These were total educational opportunities per day, frequency of challenging behaviour per day, intervention acceptability, social validity and intervention compliance. As all students received ABA tutoring, measures of total educational opportunities per day (total trials) and frequency of challenging behaviours were taken daily as part of their current educational approach. Treatment acceptability was measured using the Therapy Attitude Inventory (TAI; Brestan et al. 1999). Social validity was measured using the Treatment Evaluation Inventory-Short Form (TEI-SF; Kelley et al. 1989). Finally, compliance was measured based on the parent sleep diary.

### Data Collection

#### Parent Sleep Diary

Parents were instructed to observe and measure their child’s sleep behaviours each day using a sleep diary. This documented the time the child was bid goodnight and placed in bed, the time the child fell asleep, final awakening time, number and duration of night wakings, total sleep duration, frequency and duration of naps, bedtime non-compliance, co-sleeping and confidence in intervention implementation. It also included an open ended ‘notes’ section within which parents were requested to record extraneous factors which may have impacted their child’s sleep (e.g. illness, absence of a parent, etc.). The diary was split into 7 days with each day commencing at 10 a.m. and ending the following day at 10 a.m. (e.g. Mondays recording commenced at 10 a.m. on Monday). Though the sleep diary was similar for both conditions, the bedtime fading condition included a section in which the bedtime for that night was inscribed by the researcher.

### Pre-intervention Measures

#### Sleep Assessment and Treatment Tool (SATT, Hanley 2005)

The SATT is a functional assessment interview which aims to identify the relation of environmental variables to sleep problems. The interview consists of sections such as sleep history, goals, problem identification, antecedent conditions, consequent actions, sleep schedule and interfering behaviour.

#### Children’s Sleep Habits Questionnaire (CSHQ, Owens et al. 2000)

Parents were asked to complete the CSHQ based on their child’s current sleep behaviours. This is a 45 item parent-completed questionnaire that seeks to evaluate child sleep problems. Questions extend over domains such as bedtime behaviour, morning waking, daytime behaviour and sleep duration. Questions are scored using a five point frequency Likert scale from never to always. A score of over 41 is indicative of a sleep problem.

### Post-intervention Measures

#### Therapy Attitude Inventory (TAI; Brestan et al. 1999)

The TAI is used to measure parent satisfaction of behavioural interventions for their children. It consists of ten questions answered using a five point Likert scale.

#### Treatment Evaluation Inventory-Short Form (TEI-SF Kelley et al. 1989)

This seeks to assess parental perceptions of treatment acceptability and efficacy. This consists of nine items scored using a five point Likert scale which moves from strongly disagree (1) to strongly agree (5). Total scores can range from 9 to 45 with a higher score representing greater acceptance.

### Experimental Design

This research employed a concurrent multiple-baseline across subjects design.

Within multiple baseline designs the intervention is implemented for the first participant while baseline conditions are maintained for other participants.

Once criterion is reached for the first participant the intervention is implemented for the following participant. Maintaining baseline conditions until criterion is reached allows for replication of results across participants (i.e. if participant 2 also demonstrates success we can consider that a replication of participant’s 1 results). Additionally it allows us to validate our prediction, as if responding remains constant for participants across baseline and changes only following intervention internal validity increases. As there were three primary dependent variables of interest three multiple-baselines were conducted for each of the independent variables (bedtime fading and positive routines).

### Interobserver Agreement (IOA)

Total duration interobserver agreement (IOA) was conducted for the purpose of reliability. This was assessed by having both parents independently observer and score the child for each of the primary dependent variables during baseline and intervention. This has been found an effective strategy for sleep research within the home setting (Mindell et al. 2006). IOA was calculated by dividing the shorter duration by the longer duration and multiplying this by 100. IOA was calculated for 42% of nights and was 100% (range 100–100) both at baseline and intervention. Mean IOA was 100% (range 100–100) for total sleep duration, 100% (range 100–100) for sleep onset latency, and 100% (range 100–100) for night wakings.

### Procedure

#### Pre-baseline

Before participant recruitment commenced consent was obtained from the director of education to consent to participant recruitment within the centre. Prior to baseline a preliminary meeting was held by the researcher with each set of parent participants individually. Within this meeting basic information regarding the child’s sleep difficulties was obtained to omit medical concerns. Following this, parents were informed about the study’s purpose and procedures. Informed consent was obtained and questions answered. The SATT and CSHQ were also completed. Parents were provided with the sleep diary and instructed to commence baseline recording.

#### Baseline

Within baseline parents were asked to continue current routines and sleep practices. An instruction sheet detailing how to measure dependent variables was provided.

#### Intervention

Once stable baseline had been obtained for all participants, interventions were implemented for the first participants of both interventions. The precise procedure for each is outlined below.

#### Bedtime Fading

##### Stage 1

Following stable baseline responding by all participants parent training was provided to the first parent participants. This consisted of detailed explanations of the intervention, modelling, role play and written instructions. Following training parents were instructed to commence implementation. Baseline measurement continued to be taken for all other participants.

##### Stage 2

Intervention implementation was commenced for participant 1. The intervention is a partial replication of Piazza and Fisher (1991). Baseline data were used to establish initial bedtime. This was done by calculating the average sleep onset time and adding 30 min. Parents were advised to prevent sleep prior to scheduled bedtimes and ensure the child woke at the prescribed wake time. Target sleep onset latency was set at 15 min. If the child demonstrated a sleep onset latency of 15 min or less bedtime was faded earlier by 15 min the following night. This is a departure from Piazza and Fisher (1991) who reduced bedtime by 30 min. It is consistent with more recent studies which posit gradual fading as more successful (Ashbaugh and Peck 1998). If the child failed to demonstrate a sleep onset latency of 15 min, bedtime was moved later by 30 min the following night. This was continued until the target bedtime for the participant was met. Target bedtime was based on developmental norms and parental wishes. Target bedtimes for each participant are outlined below (Table 2). As per Piazza and Fisher’s protocol (1991) parents were advised that if their child woke during the night they were to respond as per baseline with the addition of vocal verbal prompts every 30 min to return to bed. Baseline data continued to be taken for participants 2 and 3.

**Table 2:** Target sleep/wake times

Participants	Target bedtime	Target wake time	Total target sleep duration
Niamh	9 pm	7.30 a.m.	10.30
Mary	9 pm	7.30 a.m.	10.30
Thomas	8.30 pm	7 a.m.	11.30

##### Stage 3

Once an increase of total sleep duration of 1 h and a sleep onset latency of below 15 min was demonstrated by participant 1 for two consecutive nights the intervention was implemented for participant 2 (stage 1 and 2 of this procedure).

##### Stage 4

Following two consecutive occurrences of the target bedtime by participant 2 the intervention was implemented for participant 3 (stage 1 and 2 of this procedure).

#### Positive Routines

##### Stage 1

Similarly to the bedtime fading intervention, stage 1 for positive routines commenced with parent training for child participant 1. This consisted of a detailed description of the intervention, modelling and role play. Parents were instructed to devise a routine which could be followed each evening prior to placing the child in bed. The following guidelines were provided (Durand 1998):

1. The routine should be 30 min in duration and occur prior to bedtime.
2. Sleep readiness activities such as dressing, washing and story time should be included.
3. The order and timing of activities must be consistent each night.
4. Avoid activities which may provoke challenging behaviour.
5. Screen time should be avoided.
6. Do not extend the routine (e.g. one more turn, one more minute etc.).

Parents were instructed to select a bedtime at which their child would have a high probability of sleep and maintain this time.

##### Stage 2

Following parent training the intervention was implemented for participant 1. The intervention is based on Christodulu and Durand (2004). Parents were provided with a visual schedule containing pictures of each step in the routine. Parents were instructed to commence the intervention by presenting the visual schedule to the child and telling them it’s time to get ready for bed. Parents should then point to the first picture on the routine and inform their child of the activity to take place. Upon completion of the activity the child should receive social positive feedback. This should continue until all steps in the routine had been completed. Following this the parent placed the child in bed, bid them goodnight and vacated the room. If at any stage the child emitted challenging behaviour (tantrums, self-injurious behaviour, etc.) the routine was to be terminated and the child placed in bed immediately. Parents were instructed that if their child awoke during the night, they were to intervene with a vocal verbal prompt every 30 min to return to bed. Further co-sleeping was not permitted. Baseline recording continued for participants two and three.

##### Stage 3

Once stable responding had been demonstrated by participant 1, the intervention was implemented for participant 2 (stages 1 and 2 above).

##### Stage 4

As stage 3, once stable responding was demonstrated by participant 2, the intervention was implemented for participant 3 (stages 1 and 2 above).

#### Post-experimental Measures

Upon intervention completion, parents attended a final meeting with the researcher. Within this meeting post-experimental measures were completed. In order to assess whether either intervention resulted in socially meaningful results a social validity questionnaire was completed by the parents (TEI-SF). Parents also completed the TAI.

## Results

### Pre-baseline Measures

#### Sleep Assessment and Treatment Tool (SATT)

The SATT was completed prior to baseline. Results of the SATT indicated all children exhibited difficulties with sleep onset. Niamh, Mary, Martin, Thomas and Alan also demonstrated early awakenings. Martin met criteria for sleep interfering behaviour. All parents noted shorter sleep onset latency and longer total sleep duration as primary goals for their children. In addition, Mary, Niamh and Martin’s parents referenced a later wake time as a goal for their child. None of the parent participants felt they had a stable bedtime routine.

#### Child Sleep Habit Questionnaire (CSHQ)

The CSHQ was completed by each set of parent participants prior to baseline recording. A score of 41 is indicative of a sleep disorder. All but one participant obtained scores above 41 (mean score = 46.5, range 37–67). Participant scores are outlined in Table 3.

**Table 3:** SATT data for participants

Intervention	Positive routines			Bedtime fading		
Participants:	Martin	Alan	John	Mary	Niamh	Thomas
Bedtime resistance	12	13	6	14	13	6
Sleep onset delay	3	1	2	3	3	3
Sleep duration	3	7	5	4	3	4
Sleep anxiety	6	8	8	6	4	4
Night wakings	3	7	7	6	6	4
Parasomnias	9	9	6	7	6	7
Sleep disordered breathing	5	7	4	1	2	1
Daytime sleepiness	8	15	3	8	7	8
Total score	45	67	41	48	41	37

### Bedtime Fading

#### Sleep Onset Latency

**Fig. 1:**
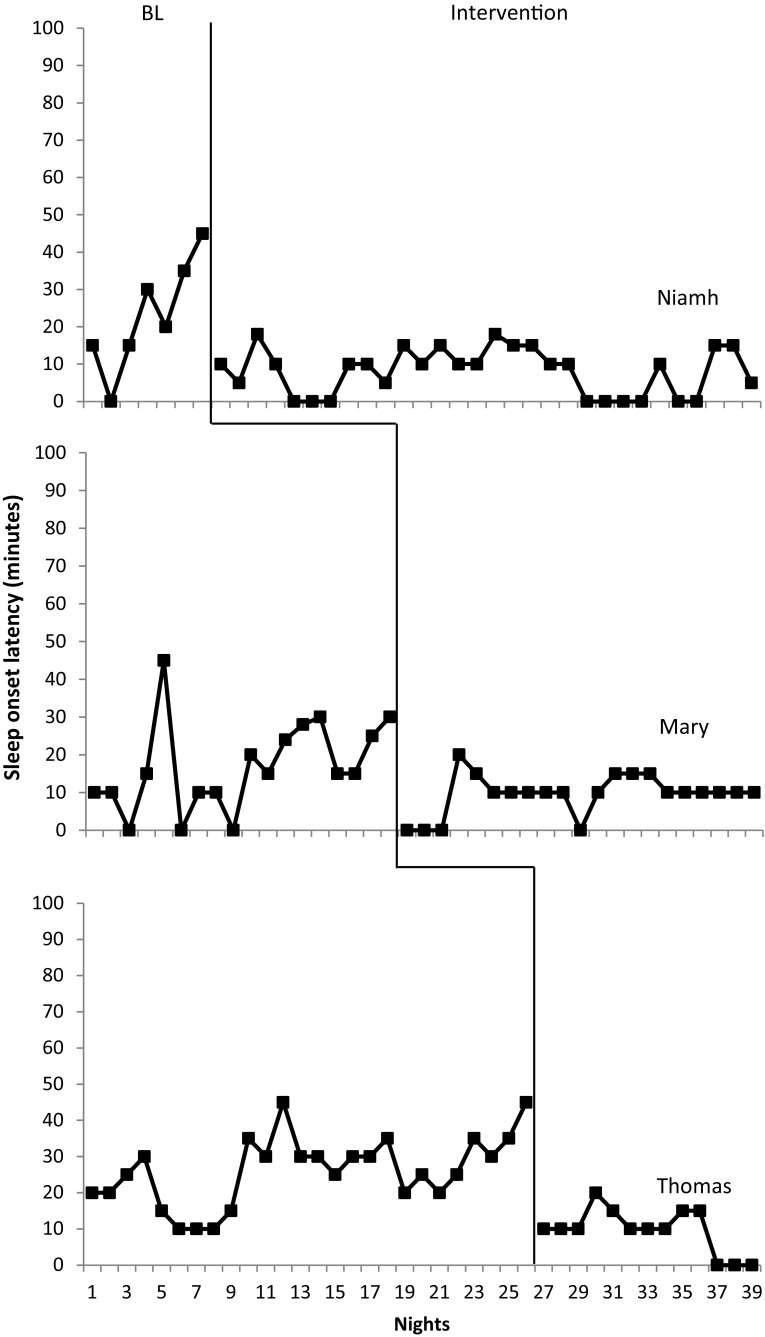
Sleep onset latency in minutes across nights for bedtime fading

The impact of bedtime fading on sleep onset latency is depicted in Fig. 1. Prior to intervention, sleep-onset latency was highly variable for all participants. Mary demonstrated a mean sleep onset latency at baseline of 16.18 min with a range of 0–45 min. Niamh also exhibited variability (mean = 22.45 min, range 0–45 min). Thomas demonstrated a narrower range of latencies (10–45 min, mean = 26.15 min). Intervention implementation for all participants occurred following increased sleep onset latency rather than stable responding. Following intervention an immediate decrease in both duration and variability of sleep onset latency was observed for all participants. For Mary mean sleep onset latency dropped to 9.52 (mean decrease = 7.26) with a range of 0–20 min. Similar was noted for Niamh (mean decrease = 14.15, range 0–20 min). Thomas’s mean also decreased to 9.61 (mean decrease = 16.55) with a similar range of 0–20 min. A high percentage of sleep onset latencies met or fell below target level (Mary = 96%, Niamh = 95%, Thomas = 94%) in comparison to baseline (Mary = 73%, Niamh = 33%, Thomas = 32%). Though decreased sleep onset latency was immediate for all participants Niamh exhibited an increase in sleep onset latency outside the target range within the first week of intervention (20 min). Sleep diary records noted Niamh had stayed with grandparents who had not ran the intervention.

#### Night Wakings

For all participants within this condition night wakings occurred at a low rate both at baseline and following intervention. All three participants exhibited only one night waking each during baseline (mean duration = 45 min, range 15–45 min). Following intervention no night wakings were observed for Thomas and Niamh. Though Mary exhibited one night waking of relatively long duration (150 min) her parents reported that she was ill and had woken for this reason.

#### Total Sleep Duration

**Fig. 2:**
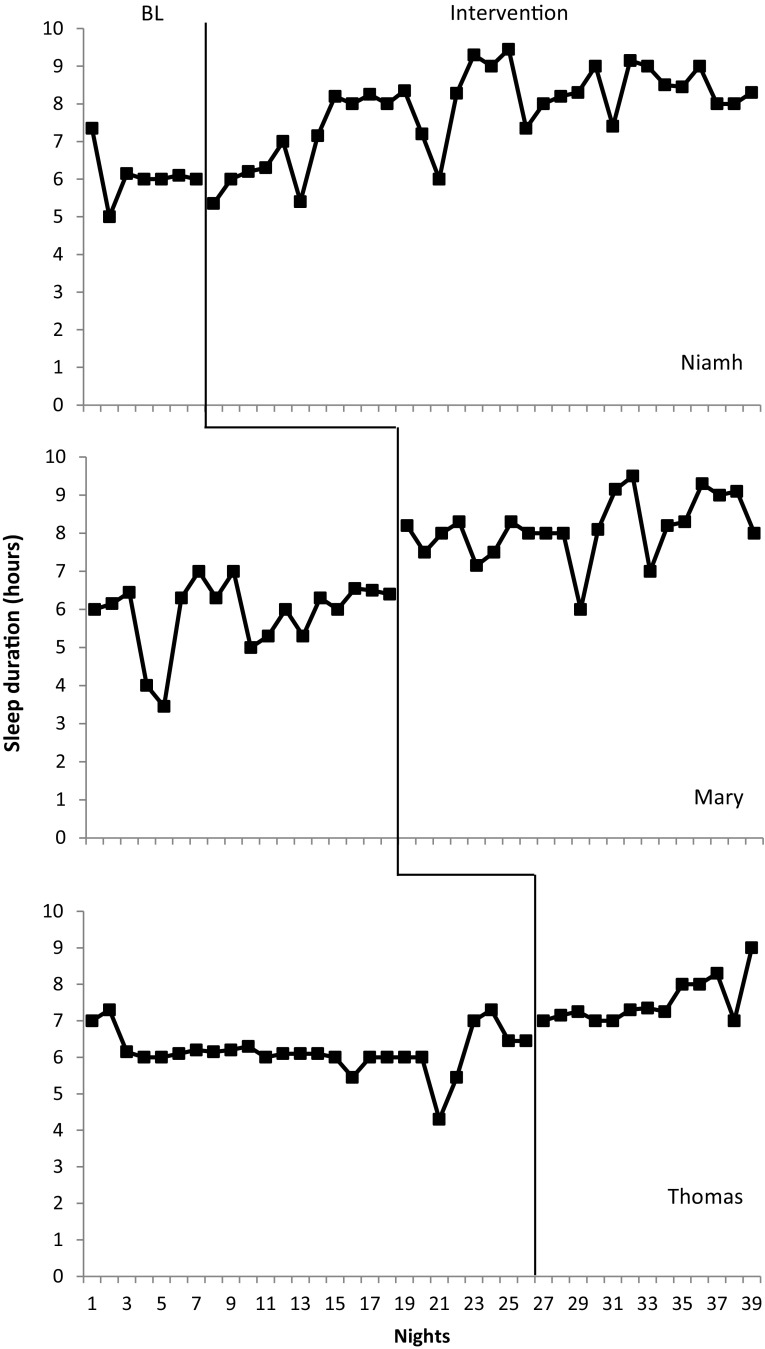
Total sleep duration across nights for bedtime fading

Total sleep duration data were obtained by measuring the total duration from sleep onset to final sleep offset minus night wakings. Results obtained are displayed in Fig. 2. Both Niamh (mean = 6 h 8 min, range 5–7.30) and Thomas (mean = 6 h 15 min, range 4.30–7.30) demonstrated consistent baseline total sleep durations. Mary exhibited variability in her sleep (mean = 5 h 40 min, range 3.45–7). Following stable baseline responding the intervention was implemented. Mean total sleep duration increased for all participants following intervention. For Niamh her mean increased to 7 h 50 min (mean increase = 1 h 42 min), Mary demonstrated a mean of 8 h 2 min (mean increase = 2 h 40 min) and Thomas exhibited a mean of 7 h 59 min (mean increase = 1 h 44 min). As such, all participants noted an increase in their total sleep duration. Though Mary’s increase was largest responding remained variable. Niamh’s graph also demonstrates some variability. All three graphs demonstrate an upward trend following intervention. This is particularly notable for Thomas and Niamh. Though mean total duration of sleep increased for all participants, target sleep duration was not met by participants (10 h 30 min for Mary and Niamh and 11.5 h for Thomas).

### Secondary Dependent Variables

#### Educational Opportunities per Day

Mean pre-intervention learn units were 107.9 with variability across participants (range 66.4–137.25). The number of learn units per day increased following intervention for all participants (mean increase = 4.42, range 0.9–7.35) (Table 4).

**Table 4:** Secondary dependent variables data for bedtime fading

	Learn units per day		Challenging behaviour		PSI-SF
	Mean	Range	Mean	Range	Mean
Niamh					
Baseline	120	70–167	28.9	20–30	105
Intervention	125	62–171	11.7	0–25	102
Mary					
Baseline	137.25	123–155	9.29	0–27	109
Intervention	144.6	22–213	1.25	0–7	100
Thomas					
Baseline	66.4	67.3	0.04	0–3	78
Intervention	58.90	43–89	0	0	78

#### Challenging Behaviour

Niamh and Mary both exhibited high levels of challenging behaviour with mean baseline frequencies of 28.9 and 9.2 respectably. Thomas exhibited near zero rates with only 1 day containing challenging behaviour. Behaviour frequency following intervention implementation decreased for all participants (mean = 8.42, range 0.04–17.2) (Table 4).

### Positive Routines

#### Sleep Onset Latency

**Fig. 3:**
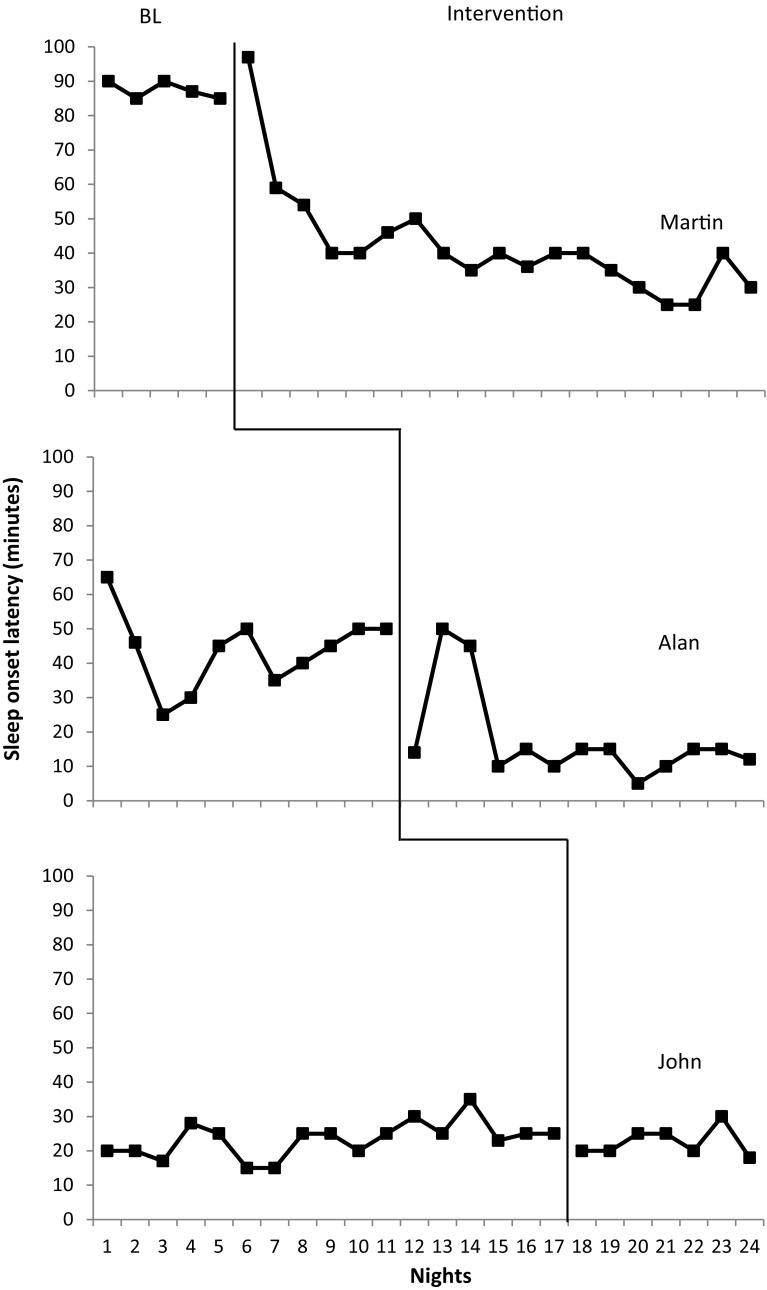
Total sleep onset latency in minutes across nights for positive routines

Sleep onset latency data are presented in Fig. 3. Martin presented with the greatest baseline mean sleep onset latency at 87.4 min with minimal variability (range 90–85 min). Alan also presented with a long sleep onset latency (mean = 44.6) and high variability (range 25–65 min). The lowest mean at baseline for sleep onset latency was observed for John (mean = 23.4 min, range 15–30 min). The intervention was implemented for all participants following stable baseline responding. Following intervention, mean sleep onset latency decreased for all participants. Martin exhibited the greatest mean decrease at 45.19 min with a mean sleep onset latency of 42.21 min following intervention. Variability of responding remained high with a range of 30–97 min. Alan also exhibited a much lower mean following intervention at 17.59 min (mean decrease = 25.96). John exhibited a decreased mean (22.57 min) however this decrease was minimal (0.54 min). Results suggest decreases occurred at a fast rate for both Martin and Alan. Both participants exhibited a sharp decrease in latency within the first 4 days of intervention. Though target sleep onset latency (sleep onset within 15 min) was not consistently reached, Alan increased the percentage of nights on which the target sleep onset latency was reached (baseline = 0%, post-intervention = 92%). Martin however did not meet target sleep onset latency following intervention but his data present a sharp descending tendency. A decrease in the percentage of target sleep onset latencies achieved was noted for John following intervention (baseline = 11%, post-intervention = 0%).

#### Total Sleep Duration

**Fig. 4:**
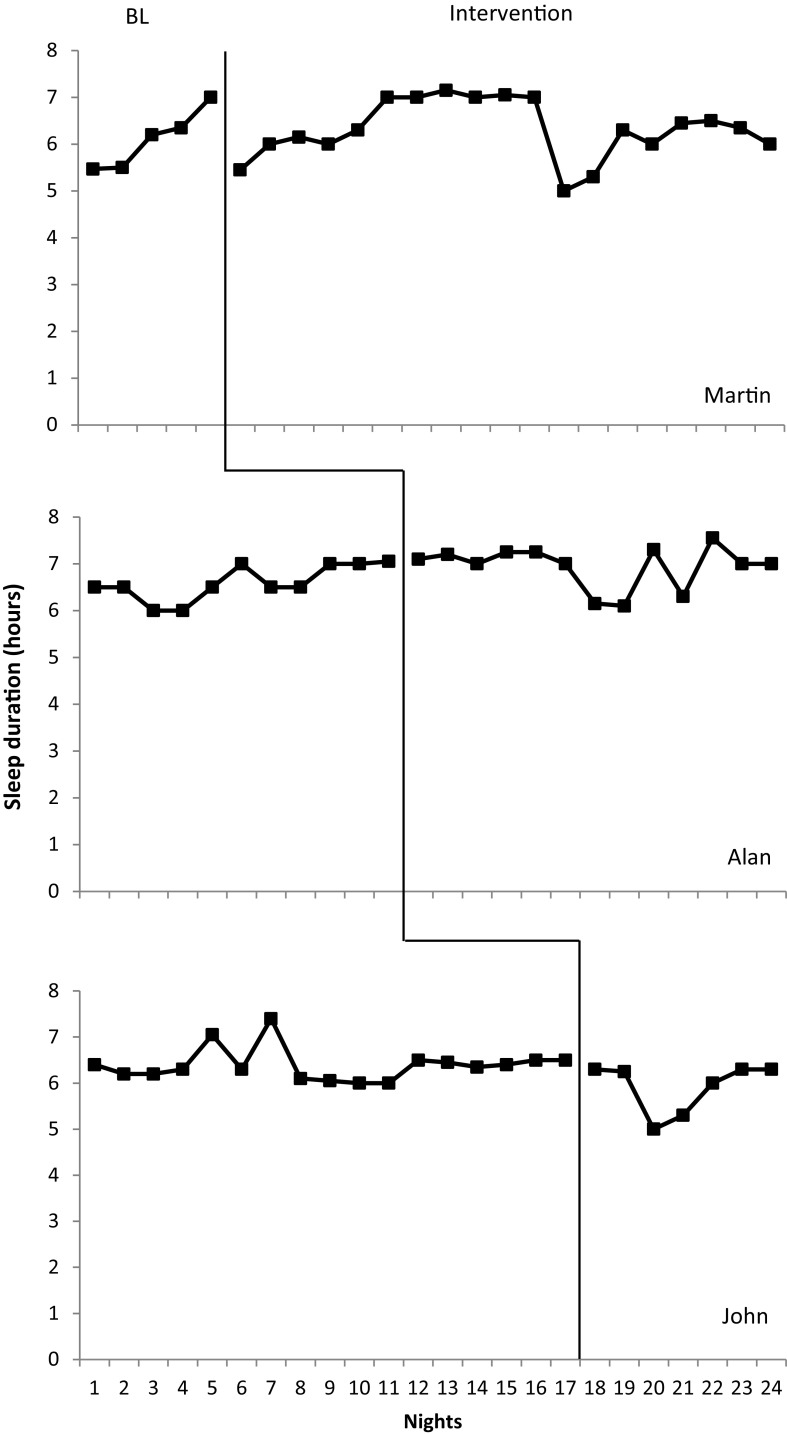
Total sleep duration across nights for positive routines

The results obtained for total sleep duration for the positive routines intervention is displayed in Fig. 4. All participants demonstrated low baseline total sleep durations. Martin presented with the lowest mean total sleep duration at 6.1 h (range 5.47–7 h). However this was minimally less than John (mean = 6.4, range 6–7.40 h) and Alan (mean = 6.39, range 6–7.05). Following intervention, an increase in mean total sleep duration was noted for both Alan (6.59 h) and Martin (6.3 h). However increases were minimal (mean increase = 0.20 for both). It should also be noted that though the mean duration increased, greater variability was noted for Alan (6.3–7.25). John differed greatly from other participants in that his total sleep duration decreased following intervention (mean = 5.9, mean decrease = 0.5). Target total sleep duration was not met by any of the participants within the positive routines intervention.

#### Night Wakings

**Fig. 5:**
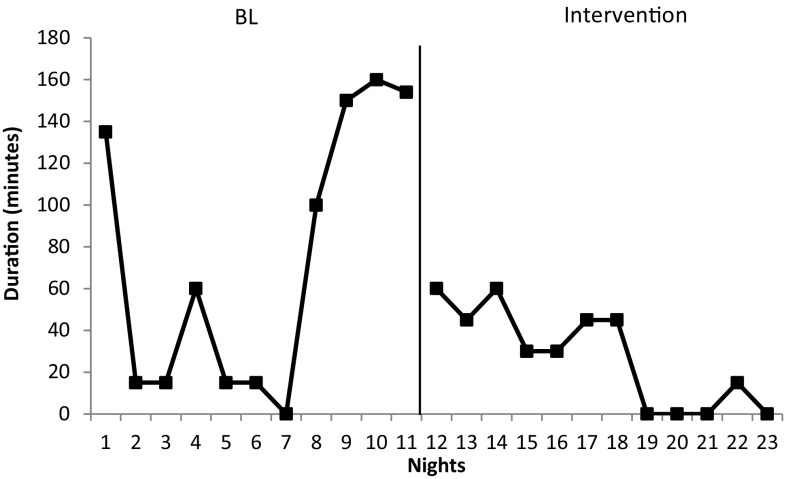
Total duration of night wakings in minutes across nights for Alan

Night wakings were not observed for John or Martin during baseline data recording. Frequency of night wakings for Alan remained consistent with data reporting one waking per night (Fig. 5). An increase in night waking duration prior to intervention implementation was noted. Mean duration of night wakings at baseline were high at 78 min (range 0–160 min). Following intervention the duration of night wakings decreased to a mean duration of 27.50 min (range 0–60). This represents a mean decrease of 50.50 min. The frequency of nights with no wakings also increased post-intervention from 8 to 33.3%.

### Secondary Dependent Variables

#### Educational Opportunities per Day

Mean learn units per day are outlined in Table 5. Both Alan and Martin demonstrated increased mean learn units per day following intervention implementation. John’s demonstrated no change (mean difference = − 0. 2%).

**Table 5:** Secondary dependent variable data for positive routines

	Learn units per day		Challenging behaviour		PSI-SF
	Mean	Range	Mean	Range	Mean
Alan					
Baseline	145	110–168	0.05	0–3	81
Intervention	147.1	102–180	0.03	0–2	81
Martin					
Baseline	224.8	60–334	0	0	78
Intervention	233.2	67–150	0	0	74
John					
Baseline	187.5	154–189	1.2	0–15	103
Intervention	187.3	156–195	3.4	0–7	103

#### Challenging Behaviour

Challenging behaviour only occurred for two of the three participants. Alan’s frequency of challenging behaviour decreased following intervention by 60% (mean difference = 0.02). John’s results differed in that his rate of challenging behaviours increased following intervention by 55% (mean difference = 2.2). Parents reported John was ill when the intervention was implemented and this may have contributed to the increase observed (Table 5).

### Treatment Fidelity

Treatment Fidelity data were obtained by having parent participants record sleep data independently using two sleep diaries. For those in the bedtime fading condition high levels of treatment fidelity were noted for all participants (Niamh = 96%, Mary = 97.4%, Thomas = 100%). Similar was noted for those in the positive routines condition. Parent completed sleep diary data showed treatment fidelity at 100% for both Alan and Martin and 91.3% for John.

### Post-intervention Measures

Post-intervention measures included the TEI-SF and the TAI (Table 6).

**Table 6:** TEI-SF and TAI data

Intervention	Participant	TEI-SF	TAI
Bedtime fading	Mary	42	50
	Niamh	40	49
	Thomas	41	44
Positive routines	Alan	43	49
	Martin	43	47
	John	37	49

#### Social Validity

Social validity data were obtained using the TEI-SF, a 10-item 5-point likert scale measure with higher scores reflecting greater social validity. The mean social validity measure for all participants was 41 (bedtime fading = 41, positive routines = 41). Scores did not vary greatly across participants with a range of 39–42 noted.

#### Treatment Acceptability

Treatment acceptability was recorded using the TAI, a 10-item 5-point likert scale with higher scores reflecting greater acceptability. All participants scored the TAI highly with a mean score of 48 (bedtime fading = 47.7, positive routines = 48.33). Differences in acceptability across interventions were minimal with positive routines scoring 0.63 above bedtime fading.

## Discussion

This study sought to evaluate the impact of two stimulus control sleep interventions on total sleep duration, night wakings and sleep onset latency for young children with ASD. The interventions investigated were positive routines and bedtime fading.

Results obtained suggest the efficacy of parent implemented bedtime fading for young children with autism. Bedtime fading was found to increase total sleep duration and decrease sleep onset latency for all participants. As minimal night wakings were exhibited no conclusions can be made on intervention effects. Secondary dependent variable measures also demonstrated increased learning opportunities and decreased challenging behaviour following intervention. Results obtained for positive routines note decreased sleep onset latency for all participants following intervention implementation. Total sleep duration was found to increase for two of the three participants. However these effects were minimal. Both interventions were also found to have high social validity, treatment fidelity and acceptability by parent participants.

Results are consistent with those of Piazza and Fisher (1989, 1991). This study expanded on these findings by demonstrating efficacy within the home environment using parents as active change agents. Further, Piazza and Fisher (1989, 1991) examined bedtime fading within a multicomponent intervention whereas this study demonstrated efficacy as individual interventions. It should be noted that though participants demonstrated increased total sleep durations, none met target sleep durations within the study. However as Sadeh et al. (2003) noted an increase of only 30 min can significantly impact a child’s daily behaviours. As such, the gains made are socially significant for participants and were further confirmed by results obtained in the TEI-SF and TAI. Total sleep duration may have been hampered by the strict sleep/wake times which form the intervention. Decreased frequency of challenging behaviour is also consistent with previous research (Piazza et al. 1991).

Though the results obtained support the efficacy of bedtime fading as an independent intervention for young children with ASD, positive routines results were not as robust. Introduction of the positive routines intervention did result in decreased sleep onset latencies for all participants. However, target sleep onset latencies were not consistently met and mean latencies remained high. It should be noted that baseline sleep onset latency was higher within positive routines than bedtime fading. This differs somewhat from Mindell et al. (2009) who noted decreased sleep onset latency (to target levels) following positive routines implementation for typically developing young children. The lack of an ASD diagnosis for participants within Mindell et al. (2009) study however may account for the discrepancies in results obtained. Total sleep duration data also present variability. Though duration increased for both Martin and Alan, John noted a small decrease. However John’s parents reported that he was ill throughout the intervention and that this may have impacted sleep. These results somewhat support the findings of Milan et al. (1981) and Adams and Rickert (1989) who found positive routines effective in increasing total sleep duration within the home environment. These studies however also included a bedtime fading element. This may account for the slight difference in results obtained and prevents direct comparison of effects. Night wakings data were only available for Alan with neither Martin nor John exhibiting any night wakings during the implementation of the study. Decreased frequency and duration of night wakings were noted with the decrease in duration occurring quickly. As results are not conclusive and no previous studies have evaluated the impact of positive routines on night wakings this area is particularly in need of further empirical analysis.

Secondary dependent variables also present mixed results for positive routines. No notable change to challenging behaviour was noted. With regards to educational units both Martin and Alan reported minimal increases with no change noted for John. However this is to be expected considering the lack of effects on primary dependent variables. While positive routines appear somewhat effective it is clear that further empirical analysis is warranted to fully understand its effects as an individual intervention rather than as a one of several components within an intervention package (Mindell et al. 2006).

The behavioural principles underlying both interventions may shed light on these differing results. The key assumption of a stimulus control approach to sleep is that for consistent sleep to occur, steps in the behavioural chain must come under stimulus control of appropriate discriminative stimuli (Bootzin 1977). While positive routines rely on stimulus control as the central mechanism of behaviour change, bedtime fading also utilises motivating operations. Research has noted the role of establishing operations (EOs) in sleep onset (Michael 1982). EOs such as quality, duration and time since previous sleep all alter the potency of sleep as a reinforcer. Bedtime fading functions by placing the child in bed at a time at which sleep onset is hypothesised to be quick. Pushing bedtime later may have had both value altering (sleep becomes a more potent reinforcer) and behaviour altering effects (increased likelihood of engaging in sleep appropriate behaviours). As such, the use of EOs within bedtime fading may have positively impacted intervention effects. Future research should further examine the role of EOs within sleep interventions.

This study further adds to existing research by demonstrating that slight intervention alterations can be made without impacting efficacy. For example, within the bedtime fading intervention the response cost element was removed. In previous studies had the child remained awake in bed for 15 min following the parent bidding them goodnight and leaving the room a response cost procedure was implemented (Piazza et al. 1997). This study differed in that response cost was omitted entirely. This was consistent with DeLeon et al. (2004) who noted bedtime fading as effective in the absence of a response cost element. A second slight alteration within this study was that bedtime was faded by only 15 min per night if the child demonstrated short sleep onset latency. This differed to Piazza and Fisher (1991) and Ashbaugh and Peck (1998) who faded bedtime by 30 min. This alteration may have increased the nights required to meet target sleep/wake times. This may in turn have prevented participants reaching target sleep durations within the timeframe of the research. Positive routines also permitted alterations in that routine content and duration was individualised to match child preferences and family wishes. Visual schedules were used to facilitate child participants transitioning through stages on the schedule. This differed from Mindell et al. (2009) and Christodulu (2000) where no visual supports were used.

Though the results of this study support positive routines and bedtime fading for young children with autism, limitations are apparent. First, the absence of objective measures of sleep present. The primary method of data collection was parent completed sleep diary. Research notes diaries have high validity, internal consistency, and agreement with objective measures (Mindell et al. 2006). Their self-report nature however presents caveats to results obtained. Participant bias can occur with self-report (Wade et al. 2007). Sleep diaries only contain data of which parents are aware (Sadeh 1994). As such total sleep duration may have been overestimated as parents would have been asleep, thus unable to record data. Night wakings may have been underestimated for similar reasons (Ashbaugh and Peck 1998; Sadeh 2011). Schreck and Mulick (2000) however noted that although parents may underestimate wakenings those missed cannot be considered problematic as they omit overt signals of wake (e.g. crying, calling out, etc.).

Self-report may have also impacted IOA data reliability. While parents may have influenced each other during data collection, there are no data indicating this. Future studies could use videotaping or digital devices registering physiological activity as an alternative method of collecting IOA data. Sleep diaries do present a number of benefits. The primary benefit was inclusion of data on events outside the bedroom (e.g. illness, staying in grandparents, etc.). This was valuable in terms of identifying extraneous variables which may have impacted adherence and efficacy. Future research should investigate the use of more objective measures of sleep and the reliability, accuracy and validity of parent-completed sleep diaries.

A further limitation in this study is the use of the CSHQ (Owens et al. 2000). Recent research completed by Johnson et al. (2016) noted inconsistent internal consistency ratings across subsets of the CSHQ. Additionally they raised queries on the applicability of the subsets to individuals with ASD. However as the CSHQ was used to establish the degree of severity of the sleep difficulties rather than as a pre/post measure its use within this study appeared warranted. It is clear that further research into the CSHQ is required to fully establish its effectiveness.

Inclusion criteria present a further limitation. To enrol, participants were required to be aged 2–7, sleep less than 7 h per night, present with a sleep difficulty on the SATT and have ASD. Inclusion criteria may not have been stringent enough and may have impacted generalisability. Firstly no reference to severity of ASD diagnosis or IQ was made. Though sleep disturbances are common across the spectrum, functioning level is pivotal when analysing behavioural interventions (Williams et al. 2004). Children with ASD who are non-verbal may require additional supports or prompting to adhere to interventions while higher functioning children may not. Though level of functioning was referenced by including developmental age (derived from VB-MAPP) this may not have been sufficiently precise.

A final limitation was the absence of function based interventions. Participants were randomly allocated to an intervention regardless of SATT or CSHQ scores. Further functional relations surrounding sleep were not investigated. Functional assessments are the hallmark of behavioural intervention (Hanley et al. 2003). Sleep problems have been exempt from such analyses though they represent a severe challenge for child and family. Though effective interventions have been documented, conditions under which each should be applied have not been established. Jin et al. (2013) reported the efficacy of individualised function based sleep interventions for young children. The SATT was utilised to provide the researchers with a hypothesised function of the sleep competing behaviours. The lack of environmental contingency evaluation of participants sleep difficulties may have adversely impacted results. Future research should examine the functional relations and use these to guide intervention.

These results impact current clinical approaches in a number of ways. The apparent efficacy of stimulus control techniques is beneficial as it minimises the need to utilise extinction based protocols which often have multiple side-effects (Jin et al. 2013). Reid et al. (1999) noted a drop-out rate of 20% of parent participants from extinction based interventions due to a reluctance to ignoring crying. Stimulus control interventions in comparison do not result in prolonged periods of crying or increased rates of challenging behaviours (Burke et al. 2004). No parent participants within this study noted increased challenging behaviour associated with sleep related stimuli. Further challenging behaviour decreased for participants within the bedtime fading intervention. A key advantage of stimulus control interventions is the facilitation of skill acquisition where extinction does not permit this (Cortesi et al. 2010). Finally, intervention implementation at the child’s bedtime is advantageous. At this time parental fatigue is minimised increasing implementation fidelity (Kodak and Piazza 2008). The high social validity, treatment fidelity and acceptability scores obtained within this study also support the positive effects of implementing sleep interventions at this time. This suggests that future research on stimulus control interventions rather than extinction based approaches is warranted.
